# Emotional Intelligence and Self-Perception in Adolescents

**DOI:** 10.5964/ejop.v14i3.1506

**Published:** 2018-08-31

**Authors:** Alejandra Daniela Calero, Juan Pablo Barreyro, Irene Injoque-Ricle

**Affiliations:** a Consejo Nacional de Investigaciones Científicas y Técnicas (CONICET), Facultad de Psicología, Universidad de Buenos Aires, Buenos Aires, Argentina; Department of Psychology, Webster University Geneva, Geneva, Switzerland; Institute of Psychology, University of Wroclaw, Wrocław, Poland

**Keywords:** emotional intelligence, self-concept, self-esteem, adolescence

## Abstract

Emotional intelligence includes self-perception regarding attention to feelings, clarity of feelings and mood repair. The aim of this work is to study the relationship between emotional intelligence, self-concept, and self-esteem. The sample included 137 adolescents from Buenos Aires City, that attended middle school, with a mean age of 13.12 years old (SD = 1.79). Correlation analysis and linear regression analysis were performed. Results showed significant positive correlations between self-esteem and clarity of feelings on the complete sample and the female subsample, and between mood repair and self-esteem on the male subsample. The linear regression analyses showed results on the same line. It´s concluded that positive self-evaluation regarding emotions, emotion comprehension and recovery can minimize the effect of negative experiences.

The term emotional intelligence was coined by Salovey and Mayer (1990). Is the ability to perceive, understand and control our emotions and those of others, and use them to guide our thoughts and emotions so that it is beneficial to the individual and the environment to which he or she belongs. Salovey and Mayer argue that it is a genuine intelligence based on the adaptive use of emotions and their application in our behaviors (Fernández-Berrocal & Extremera, 2008; Mayer, Caruso, & Salovey, 1999).

According to Salovey and Mayer (1990), perceived emotional intelligence has aspects: attention to feelings, clarity of feelings and mood repair. These aspects explain the individual differences in the way people deal with emotions. Attention to feelings refers to how people consider their emotions; clarity of feelings is how people think they understand and discriminate their feelings; and finally, mood repair is the subject's belief that he/she can modify his/her negative moods.

The model of emotional intelligence by Salovey and Mayer (1990) allows to investigate the meta-knowledge of emotional states and, above all, how these abilities can affect other areas of a person (Extremera & Fernández Berrocal, 2005). Many of the studies based on Salovey and Mayer’s model found that emotional intelligence is an important construct for the development of different cognitive, physical, and social skills (Extremera, Durán, & Rey, 2009; Extremera, Salguero, & Fernández-Berrocal, 2011; Jiménez & López-Zafra, 2008; Lopes, Salovey, & Straus, 2003; Martinez-Pons, 1997; Martins, Ramalho, & Morin, 2010; Mayer, DiPaolo, & Salovey, 1990; Salovey, Mayer, & Caruso, 2002; Salovey, Stroud, Woolery, & Epel, 2002).

In adolescence, several studies found a relationship between perceived emotional intelligence and psychological adjustment (Jiménez & López-Zafra, 2011; Salguero, Fernández-Berrocal, Ruiz-Aranda, Castillo, & Palomera, 2011; Salguero, Palomera, & Fernández-Berrocal, 2012). Furthermore, Palomera, Salguero, and Fernández-Berrocal (2011) found associations between high clarity of feelings and high mood repair with low levels of depression, anxiety and school maladjustment. Also, Fernández Berrocal and Ramos Díaz (1999) argue that the belief in being able to prolong positive moods and interrupt negative ones ensures a good level of mental health. Moreover, Palomera et al. (2011) observed a correlation between high attention to feelings with high levels of depression, when levels of clarity of feelings and mood repair are low. At the same time, Jiménez and López-Zafra (2011) found that perceived emotional intelligence correlates positively with prosocial attitudes related to social competence, such as leadership, cooperation, and social sensitivity. Furthermore, Otero Martínez et al. (2009) found moderate significant correlations between perceived emotional intelligence and academic achievement.

In relation to the differences in emotional intelligence between genders, studies show that a tendency of higher levels of emotional attention in women (Fernández Berrocal & Ramos Díaz, 1999; Lasa, Salguero, Fernández Berrocal, & Aritzeta, 2010; Sánchez Núñez, Fernández Berrocal, Montañés Rodríguez, & Latorre Postigo, 2008).

Self-concept and self-esteem are considered one of the most important variables of personal well-being (García Pérez, Musitu Ochoa, & Veiga, 2006). Harter (1988) defines self-esteem as the general level of self-evaluation. It is a general judgment of self-worth. Harter also defines self-concept as the evaluative judgment of the self, circumscribed to specific domains of life as being: physical appearance, intimate friendship, social acceptance, good behavior, school competition, job competence, and sports competition (Harter, 1988). Self-esteem is a supraordinal construct, of an order of abstraction superior to self-esteem (Harter, 1999). In fact, the assessment that a person makes of him/herself in the different domains of self-concept, and the importance that he/she attributes to these domains, influence self-esteem. Moreover, the overall judgment is determined by the degree of importance that the individual gives to success in each specific domain (Harter, 1986). Although in childhood the descriptions of the self are concrete, in adolescence reaches a more abstract level, where there is an increase in the number of domains of the self-concept (Harter, 1983). This increase is due to cognitive development and to interactions with a greater number of contexts, that allows children to gradually recognize that their attributes and behaviors vary from one context to another (Calero & Molina, 2016). Coleman and Hendry (2003) found that in adolescents these variables are linked to the psychological adjustment that the adolescent shows. On the other hand, Oliva Delgado et al. (2010) consider them as central aspects for a positive development during adolescence.

Regarding gender differences in self-concept and self-esteem in adolescence, Harter (1999) reported differences in physical appearance and sports competition in favor of males. For their part, Pastor, Balaguer, and García-Merita (2003) found differences in academic competence and global self-esteem in favor of males, with women reporting higher levels of intimate friendship.

Different studies found positive relationships between emotional intelligence and self-esteem (Cabello, Fernández-Berrocal, Ruiz Aranda, & Extremera, 2006; Salovey, Stroud, et al., 2002; Schutte, Malouff, Simunek, McKenley, & Hollander, 2002). Additionally, Matalinares Calvet et al. (2005) reported positive relationships between emotional intelligence and self-concept.

The aim of the present study is to study the relationship between emotional intelligence with self-concept and self-esteem in adolescents. On the other hand, it is sought to explore if any of the aspects of emotional intelligence has a predictive effect on global self-worth, that is, self-esteem. In addition, it is proposed to explore if there are differences in the relationship of emotional intelligence and self-esteem in both genders.

## Method

### Participants

The sample consisted of 399 adolescents (263 women -69.9%- and 136 males -34.1%-), with a mean age of 15.16 years (*SD* = 1.79), attending a high school in Buenos Aires City, Argentina. The subjects participated with the informed consent of their parents and were assured anonymity of the participation and the confidentiality of the information.

### Procedure

The administration of the instruments was done in a single session during class hours. All the instruments were delivered in closed envelope and once the instruments were completed, returned to the evaluators also in a sealed envelope.

Teachers or staff of the educational institution were prevented from attending during the administration.

### Materials

#### Trait Meta-Mood Scale

Trait Meta-Mood Scale (Calero, 2013) assesses the perceived emotional intelligence through three dimensions: attention to feelings, clarity of feelings, and mood repair. The scale incudes 21 items (seven of each dimension), with five response options (from "totally agree" to "totally disagree"). A score of each of the components of perceived emotional intelligence is obtained from the sum of each item responses. Higher scores reflect higher rates of perceived emotional intelligence. The scale shows adequate reliability indexes by internal consistency (Cronbach's Alpha): .81 (attention to feelings), .86 (clarity of feelings), and .85 (mood repair). Likewise, adequate indicators of validity were found (see Calero, 2013).

#### Self-Perception Profile for Adolescents

Self-Perception Profile for Adolescents (SPPA, Harter, 1988; Adaptation: Facio et al., 2006) is a scale that evaluates global self-esteem and self-concept across different specific domains. It consists of eight subscales of five items each (physical appearance, romantic appeal, close friendship, social competence, behavioral conduct, scholastic competence, athletic competence, and global self-worth or self-esteem). Physical appearance assesses to what extent the adolescent is satisfied with his or her appearance; romantic appeal assesses the extent to which the adolescent is considered attractive to people with whom he would be interested in having a love relationship; close friendship assesses the perception of your ability to make close friends; social competence evaluates to what extent the adolescent feels accepted by his peers; behavioral conduct assesses to what extent you are satisfied with your behavior, you believe you are doing the right thing and you avoid getting into trouble; scholastic competence evaluates how adolescents perceive their academic ability; athletic competence evaluates how the adolescent perceives his ability in sports; job competence, evaluates how the adolescent perceives himself in his ability to carry out a job; and global self-worth assesses the extent to which the adolescent likes his or her person, is happy with how he/she lives and how he/she is. It is a global value judgment. In each item, there are two contrary statements and the adolescent must choose one and then establish the degree of agreement with the chosen option. The scores of the items are summed and a score is obtained in each domain evaluated, the higher scores reflect a better self-perception. Given the sample characteristics, the job competence domain was not administered. The instrument has adequate indicators of validity. Cronbach's alpha was physical appearance .88, romantic appeal .74, close friendship .74, social competence .71, behavioral conduct .73, scholastic competence .67, athletic competence .84, and global self-esteem .75 (see Facio et al., 2006).

### Data Analysis

The Kolmogorov-Smirnov test indicates that distribution values deviate significantly from the theoretical percentiles of an asymptotic normal distribution (see Table 1). For this reason, it was decided to use statistics that have as reference non-normal parameters. To carry out the correlation analysis between the perceived emotional intelligence and the levels of self-concept and self-esteem, *Spearman’s Rho* test was used. To evaluate the predictive power of emotional intelligence factors (attention to feelings, clarity of feelings, mood repair) on self-esteem a multiple linear regression was performed with the method of least squares estimation, having previously checked the assumptions of normality, linearity, homoscedasticity, and independence of residues.

**Table 1:** Descriptive Statistics and Kolmogorov-Smirnov Normality Test

Variable	*M*	*SD*	*Kurtosis*	*Skewness*	K-S	*df*	*p*
Attention to feelings	3.64	.67	.12	-.35	.07	398	< .001
Clarity of feelings	3.39	.74	-.06	-.47	.10	398	< .001
Repair to feelings	3.57	3.57	-.11	-.45	.09	398	< .001
Self-esteem	2.93	.61	-.13	-.37	.10	398	< .001
Physical appearance	2.44	.77	-.81	-.10	.11	398	< .001
Romantic appeal	2.33	.61	-.10	.01	.07	398	< .001
Close friendship	3.20	.58	1.21	-.88	.11	398	< .001
Social competence	3.02	.51	.82	-.46	.12	398	< .001
Behavioral conduct	2.78	.59	-.23	-.28	.10	398	< .001
Scholastic competence	2.67	.58	-.25	-.13	.10	398	< .001
Athletic competence	2.65	.73	-.57	-.29	.11	398	< .001

## Results

Table 1 presents the descriptive statistics of the studied variables.

First, a correlation analysis between the components of emotional intelligence and self-perception will be carried out. Then a linear regression analysis will be conducted in order to analyze the predictive power of emotional intelligence factors on self-esteem.

Analysis of correlations between emotional intelligence components and Self-perception profile for adolescents domains yielded significant positive correlations between attention to feelings and close friendship (Rho = .16, *p* < .01). Likewise, there were significant positive associations between clarity of feelings and self-esteem (Rho = .28, *p* < .01), physical appearance (Rho = .17, *p* < .01), romantic appeal (Rho = .17, *p* < .01), social competence (Rho = .20, *p* < .01), and athletic competence (Rho = .17, *p* < .01). Finally, significant positive correlations were found between mood repair and self-esteem (Rho = .29, *p* < .01), close friendship (Rho = .16, *p* < .01), social competence (Rho = .24, *p* < .01), and behavioral competence (Rho = .19, *p* < .01) (see Table 2).

**Table 2:** Correlations Between Self-Esteem and Self-Concept and Emotional Intelligence

Variable	Rho.								
	Total sample (*n* = 399)			Females (*n* = 262)			Males (*n* = 136)		
	A	C	R	A	C	R	A	C	R
Self-esteem	.06	.28**	.29**	.12	.29**	.28**	.01	.17	.30**
Physical appearance	.00	.17*	.11	.13	.13	.05	-.15	.12	.20
Romantic appeal	.02	.17*	.03	.11	.12	-.01	-.14	.21	.09
Close friendship	.16*	.06	.16*	.08	.02	.10	.28*	.21	.31**
Social competence	.13	.20**	.24**	.13	.18*	.21*	.15	.24	.31**
Behavioral conduct	.10	.13	.19**	.12	.13	.15	.06	.18	.28*
Scholastic competence	.11	.07	.14	.14	.00	.07	.07	.13	.26*
Athletic competence	-.04	.17*	.14	.02	.14	.13	-.10	.08	.11

*Note*. Rho: Rho de Spearman; R: repair to feelings; A: attention to feelings; C: clarity to feelings.

*p < .05 Bonferroni correction. **p < .01 Bonferroni correction.

When repeating the analyzes by sex, in the case of women, significant correlations were found between clarity of feelings and self-esteem (Rho = .29, *p* < .001) and social competence (Rho = .18, *p* < .01). Between mood repair with self-esteem (Rho = .28, *p* < .01) and with social competence (Rho = .21, *p* = .001). In the case of men, significant associations were found between attention to feelings and close friendship (Rho = .28, *p* < .01), mood repair with self-esteem (Rho = .30, *p* < .01), close friendship (Rho = .31, *p* < .01), social competence (Rho = .31, *p* < .01), and behavioral conduct (Rho = .28, *p* < .01). In all cases, the correlations were from low to moderate intensity (see Table 3).

**Table 3:** Forecast Self-Esteem With Emotional Intelligence

Self-esteem	Attention			Clarity			Repair		
	β	*t*	*p*	β	*t*	*p*	β	*t*	*p*
Total	-.023	-.53	.60	.20	5.11	< .001	.21	5.90	< .001
Females	.052	.89	.38	.20	3.80	< .001	.21	4.67	< .001
Males	-.08	1.30	.20	.13	2.10	.04	.20	3.75	< .001

The regression analysis performed to study the predictive power of emotional intelligence factors on self-esteem showed that the model tested was significant [*F*(3, 394) = 25.94, *p* < .01, R2 = .16]. When analyzing the effects of each of the predictor variables, clarity of feelings (β = .20) and mood emotional (β = .21) showed a significant effect on self-esteem. Again, the same analysis was performed separately in females and males. The regression analysis in females showed that the model tested was also significant [*F*(3, 259) = 16.98, *p* < .01, R2 = .15]. When analyzing the effects of each of the predictor variables, it is observed that clarity of feelings (β = .20) and mood repair (β = .21) had a significant effect on self-esteem. Finally, the regression analysis in the male group equally showed that the model tested was significant [*F*(3, 132) = 16.98, *p* < .01, R2 = .13]. When analyzing the effects of each of the predictor variables, clarity of feelings (β = .13) and mood repair (β = .20) showed a significant effect on self-esteem (see Table 3).

## Discussion

The aim of this work was to study the relationship between perceived emotional intelligence and self-concept and self-esteem in adolescents. Furthermore, by considering the supramodal level that the self-esteem has on the domains of self-concept, we studied if any of the factors of emotional intelligence had a predictive effect on self-esteem. To this end, we performed a correlation analysis finding significant positive relationships between the aspects of emotional intelligence and the components of self-esteem, and the domains of self-concept. Then, through a linear regression analysis, we observed that clarity of feelings and mood repair predicted the functioning of self-esteem in the general sample, as well as in women and in men.

The findings showed that the clarity of feelings and mood repair associate to self-esteem and various domains of self-concept, while attention to feelings was only associated with close friendship. These results appear to be in line with the findings in previous works. On the one hand, several studies found relationships between levels of clarity of feelings and mood repair, and variables associated with personal well-being (Fernández-Berrocal, Salovey, et al., 2002; Palomera et al., 2011; Rey, Extremera, & Pena, 2011; Salguero et al., 2011). On the other hand, in the case of attention to feelings, the findings of the previous research are not univocal (Extremera, Durán, & Rey, 2007), and even this factor had an association with poor psychological adjustment indexes (Salguero et al., 2011). The positive relationship found in this study between attention to feelings and the close friendship domain of self-concept could be since adolescents who are more attentive to their emotions also attend the other's emotions. This allows an increase in the perception about the intimacy of relationships, which may lead to positive evaluation in the close friendship domain. Following this line, Salovey, Stroud, Woolery, and Epel (2002) found that attention to feelings positively correlated with greater empathy.

In addition, the relationships found between clarity of feelings and mood repair with self-concept domains linked to social relations (social competence and close friendship) are like those found in previous research where positive emotional intelligence had an association with different social abilities (Jiménez & López-Zafra, 2011; Salguero et al., 2011). Moreover, people who are emotionally intelligent are also more able to extrapolate their perception, understanding and managing skills to other’s emotions, which would lead to more satisfying interpersonal relationships (Extremera & Fernández-Berrocal, 2004). Since we cannot establish the directionality of the relationship from a correlation analysis, two possible interpretations arise: that the adolescents' perception of having higher levels of mood repair and clarity of feelings allows them to have a good self-perception in social nature domains of self-concept, thanks to the transfer of these skills to their performance in social relations, or, considering the fact that is the perception of their emotional intelligence is what is under evaluation, that a good self-perception in social domains could affect the perception of higher levels of clarity of feelings and mood repair.

Regarding the observed relationships between mood repair and behavioral conduct in the total sample and in the group of males, we hypothesize that adolescents who perceived themselves with more capacity to regulate their states of mind can have a better adjustment to the image of a balanced teenager who does not let himself be dragged by his negative moods. That is to say, if they fit the stereotype of a more emotionally stable adolescent, so does self-perception in relation to one's own behavior. Perhaps is due to gender differentiation that this relationship does appear in the group of women since they have a greater social license to show negative feelings.

In the group of males, we found that those who perceived themselves with better mood repair also had a better self-concept in the scholastic competence domain. Perhaps this is a result of the fact that their negative moods do not interfere with their performance as presented by Otero Martínez et al. (2009), although it would be necessary to investigate if there is also an association between the scores obtained and the reported level of mood repair to confirm this assumption.

The same interpretation could be valid for the relationship between clarity of feelings and athletic competence, which indicates that adolescents that perceived themselves with greater ability to catalog their emotions also reported a greater sports self-concept. That is, having a self-perception of a better ability to identify emotions may allow them to dedicate a greater share of attention focused on sports activity without having to focus on breaking down their emotions, which reflects in a better self-perception in this domain.

Salovey, Stroud, et al. (2002) found a positive association between self-esteem and the three perceived emotional intelligence factors. While Fernández-Ozcorta et al. (2013), as in this work, found that the relationship of self-esteem was only with the clarity of feelings and mood repair factors.

The fact that adolescents who have a clearer self-perception in identifying emotions and a greater ability to repair negative moods report higher self-esteem may be since the positive self-assessment of clarity and regulation of emotions allows them to minimize the effect of negative experiences (Schutte et al., 2002). According to Schutte et al. (2002), people with greater emotional intelligence will be able to face in a more adaptive way the negative events that provoke negative affections that could affect their value as a person.

Regarding the differences found between gender, we conclude that for women it is as important the clarity in perceiving their emotions as their capacity to recover for their global value as a person, whereas for men it is only important the perception to recover from negative moods. Perhaps these differences obey the role differentiation, as proposed by Gartzia, Aritzeta, Balluerka, and Barberá (2012) and Sánchez Núñez et al. (2008). In the case of women, the emotional instruction received from the family nucleus is more permeable to emotional experience, which could reflect a greater appreciation of their ability to discriminate and label emotions. On the other hand, in males, socialization influences them to avoid expressing emotions, increasing the appreciation of the regulation of negative moods (Sánchez Núñez et al., 2008). Thus, while in women it is only the perception about the emotional clarity that predicts the level of self-esteem, in men, along with emotional clarity, the perception of one's own ability to recover from negative moods acts.

Both the ability that adolescents perceive to have to catalog their emotions and the ability to repair their moods is related to central constructs during this life cycle for positive development. The positive relationships found to support the importance of emotional education, understanding that it would respond to social-emotional needs that are not being addressed in formal education (Bisquerra Alzina, 2003).

This work is not exempt from limitations, the sample corresponds to a population of adolescents from the Autonomous City of Buenos Aires. Is necessary to expand the population to increase the external validity of research. Finally, although the instruments are adapted for the local adolescent population, in particular the SPPA presents some items that would not work as expected, affecting the Alphas of some of the domains that make up the instrument.

Likewise, the possibility of analyzing the relationship between self-perception and measures that assess emotional intelligence along with measures of psychosocial adjustment would expand the conclusions found in the present work.
