# Correction of Calero et al. (2018). Emotional Intelligence and Self-Perception in Adolescents

**DOI:** 10.5964/ejop.23249

**Published:** 2026-05-29

In the published version of

CaleroA. D.
BarreyroJ. P.
Injoque-RicleI.

(2018). Emotional intelligence and self-perception in adolescents.
Europe's Journal of Psychology,
14(3), 632643
10.5964/ejop.v14i3.1506

some errors in numeric reporting have been identified. These were found during a reproducibility check conducted with a dataset provided by the authors. 

The corrections are as follows:

1. The sample size listed in the abstract should be 399 rather than 137.
2. The mean age listed in the abstract should be 15.16 rather than 13.12. This calculation excluded a single participant with a self-reported age of 5, whose entry appears likely to be an input error. This participant was nevertheless retained in the dataset for the remaining analyses.
3. In the method section, the percentage of the sample who were women should be 263/399 = 65.9% rather than 69.9%.
4. In Table 1, the standard deviation for 'Repair to feelings' should be 0.80 rather than 3.57.
5. In the paragraph below Table 3, the F statistic for the regression model in males should be 7.61 rather than 16.98. This F statistic remains statistically significant.

These errors do not affect the substantive conclusions presented in the article. The authors endorse these corrections.

